# A Rare Case of ALK-Positive Large B-Cell Lymphoma with CD33 Expression

**DOI:** 10.1155/2018/5320590

**Published:** 2018-06-11

**Authors:** Jessica Corean, K. David Li

**Affiliations:** Department of Pathology, ARUP Laboratories, University of Utah, Salt Lake City, UT, USA

## Abstract

Anaplastic lymphoma kinase-positive large B-cell lymphoma (ALK+ LBCL) is a very rare and aggressive subtype of diffuse large B-cell lymphoma characterized by *ALK* rearrangement. Immunophenotypically, the tumor cells are typically negative for common B-cell markers, T-cell markers, and CD30; however, they express markers of terminally differentiated B cells/plasma cells such as CD38, CD138, and MUM-1/IRF4. The diagnosis of ALK+ LBCL can be challenging, and often a large panel of immunostains is required to exclude other hematopoietic and nonhematopoietic neoplasms. To date, approximately 130–140 cases have been reported, but here we report the first known case of ALK+ LBCL with unusual CD33 expression.

## 1. Introduction

Anaplastic lymphoma kinase-positive large B-cell lymphoma (ALK+ LBCL) is a rare hematopoietic neoplasm accounting for less than 1% of diffuse large B-cell lymphomas (DLBCL) [1]. The entity was first described in 1997, and to date, approximately 130–140 cases have been reported in the literature [2]. ALK+ LBCL affects both adult and pediatric populations and can present with nodal and/or extranodal involvement. Unfortunately, the tumor is frequently discovered at a higher stage and has been shown to demonstrate aggressive clinical behavior, high relapse rate, and poor response to standard treatments, such as cyclophosphamide, doxorubicin, vincristine, and prednisone (CHOP) and CHOP-derived regimens [2, 3]. Histologically, the lymphoma cells resemble plasmablast and/or immunoblast and frequently show nodal sinusoidal growth patterns [4, 5].

The most important distinguishing feature of ALK+ LBCL from DLBCL and other lymphomas is the *ALK* rearrangement. ALK is a tyrosine kinase receptor of the insulin receptor superfamily and plays an important role in neural development [2, 6–8]. The majority of ALK+ LBCL harbor the *t*(2;17)(p23;q23) with fusion of the *clathrin* gene (*CLTC*) on chromosome 17q23 with the *ALK* gene on chromosome 2p23. Rare cases are associated with *t*(2;5)(p23;q35), as described in ALK-positive anaplastic large cell lymphoma [1]. *ALK* gene activation through point mutations and gene amplifications has also been described [6]. The classic *t*(2;17)(p23;q23) results in a unique restricted granular cytoplasmic staining pattern present by immunohistochemistry (IHC).

In addition to the characteristic ALK protein expression by IHC, the lymphoma has an immunophenotypic profile that includes expressions of terminally differentiated B-cell/plasma cell markers such as MUM-1, CD38, and CD138, but typically lack expression of CD20 (although weak expression has been reported) and CD30 [1–5]. EMA is typically positive in the tumor cells, and aberrant expression of T-cell markers has also been described. In contrast, myeloid lineage-associated markers such as CD13 and CD33 that are commonly expressed by myeloid neoplasms are not routinely performed as a part of lymphoma workup and have not been reported in ALK+ LBCL. However, CD33 has been reported in a high percentage of ALK+ anaplastic large cell lymphoma, rare cases of CLL/SLL, and Burkitt lymphoma [9–11]. To the best of our knowledge and based on literature review, we describe the first case of ALK+ LBCL with CD33 expression.

## 2. Case Presentation

### 2.1. Clinical History

A 54-year-old Caucasian male presented with an enlarging right neck mass in November, 2015. Fine-needle aspiration (FNA) was performed on the mass at that time which showed malignant cells consistent with squamous cell carcinoma. The patient did not have follow-up or further treatment at that time due to socioeconomic issues. His past medical history is significant for alcoholism, tobacco abuse, noninsulin-dependent type 2 diabetes mellitus, and osteoarthritis. For the next sixteen months, he reported three flares of painful neck adenopathy. He sought treatment, and short courses of antibiotics and steroids were administered each time.

In March of 2017, his latest flare of right-sided neck adenopathy did not respond to antibiotics and steroid treatment course. He presented to the Emergency Department and found to have a grossly palpable mass in the right neck. He reported no symptoms of fevers, chills, night sweats, fatigue, or weight loss. Computed tomography (CT) revealed multiple low-density cystic structures in the right neck consistent with necrotic lymph nodes. The lymph nodes ranged in size from 1.4 cm to 2.9 cm in greatest dimension. No additional masses were detected in nasopharynx, oropharynx, or larynx. At this point, the patient was admitted for further workup and management. PET-CT showed right neck hypermetabolic uptake ranging from SUV of 4.3 to 4.5, and a CT of the chest showed no obvious disease and no evidence of lymphadenopathy. Following an FNA suggestive of either an anaplastic carcinoma or a hematolymphoid neoplasm, an excisional biopsy of the neck mass was performed.

### 2.2. Pathology

**Figure 1:**
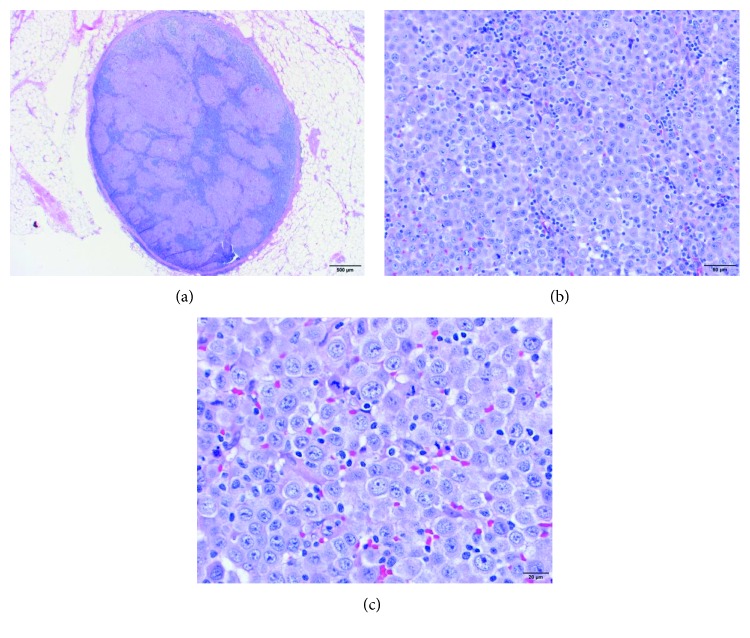
H&E sections of the biopsy specimen demonstrating an involved lymph node. Images (a), (b), and (c) are at 2x, 10x, and 40x magnifications, respectively. The tumor cells are large, monomorphic, immunoblast-like with oval nuclei, prominent nucleoli, and abundant cytoplasm. The background shows residual small lymphocytes.

Hematoxylin and eosin- (H&E-) stained right neck mass excisional biopsy material demonstrated lymph node and soft tissue with sinusoidal infiltration of large atypical monomorphic cells with round nuclei, occasional prominent central nucleoli, and abundant amphophilic cytoplasm. The lymph node was mostly effaced by tumor cells, but the uninvolved areas appeared unremarkable and showed residual small mature lymphocytes (Figure 1).

**Figure 2:**
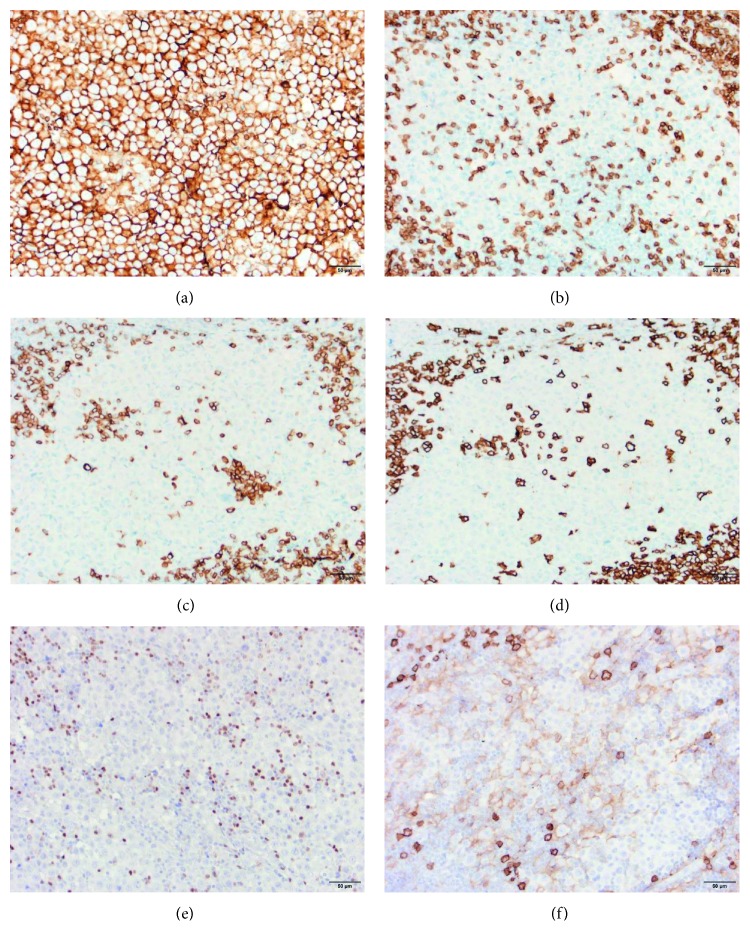
Immunohistochemical stains of the tumor cells. The tumor cells express CD45 (a) and weak CD38 (f) but are negative for CD3 (b), CD20 (c), CD79a (d), and PAX5 (e). All images are at 20x magnification.

**Figure 3:**
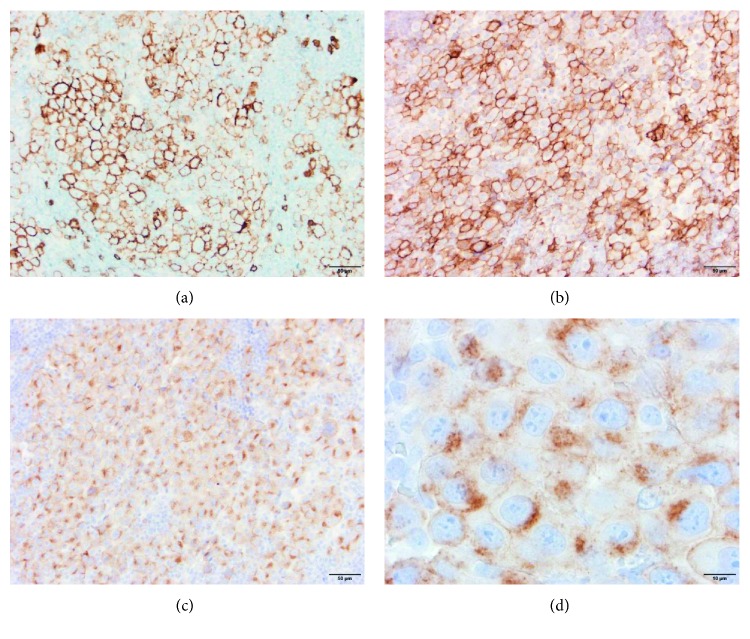
Additional immunohistochemical stains of tumor cells. The tumor cells also express CD138 (a), CD33 (b), and ALK (c). Higher magnification (100x, oil) of ALK in panel (d) demonstrates a cytoplasmic granular pattern highly suggestive of CLTC-ALK fusion protein expression. Images (a), (b), and (c) are all at 20x magnification.

An extensive immunohistochemical panel was performed to aid the diagnosis with appropriate reactive controls (Figures 2 and 3). The tumor cells expressed CD45, weak CD38, CD138, and EMA. ALK-1 showed a restricted granular cytoplasmic staining pattern highly suggestive of CLTC-ALK fusion protein expression. MIB-1/Ki-67 was approximately 80%. Interestingly, the tumor cells also showed strong and diffuse CD33 expression. The tumor cells were negative for PAX-5, myeloperoxidase (MPO), TIA-1, EBV, HHV8, CD2, CD4, CD5, CD7, CD8, CD20, CD30, CD34, CD79a, and kappa/lambda light chains. Other nonhematopoietic immunostains including CK OSCAR, CK7, melan-A, CK5/6, p40, and p63 were negative. Flow cytometry, cytogenetics, and FISH studies were not performed on the sample. Based on the morphology and immunohistochemistry, a diagnosis of ALK+ LBCL was rendered. The case was also reviewed at NIH, and the diagnosis was confirmed. Due to the aberrant CD33 expression, additional molecular studies were performed in an attempt to identify any myeloid malignancy-associated mutations. A myeloid malignancies mutation panel by next generation sequencing (NGS) was performed and did not reveal any significant mutations. Currently, the patient is under treatment protocol, which includes three cycles of CHOP chemotherapy to be followed by radiation therapy.

## 3. Discussion

ALK+ LBCL is a rare type of large cell lymphoma recognized by the World Health Organization's classification of tumors of hematopoietic and lymphoid tissue [1]. To date, less than 140 cases have been reported in the literature. ALK+ LBCL is generally considered an aggressive disease with approximately half of the reported patients dying within two years and a five-year overall survival rate of 34% [2]. Patients with stage I/II disease had significantly better overall survival at 76% and 66%, respectively [2]. Poor clinical response has been consistently reported to conventional therapies, such as CHOP and CHOP-derived treatments. New treatment options available such as ALK inhibitors have shown promising therapeutic activity in ALK+ ALCL, which could become a rationale therapy to be considered in ALK+ LBCL.

The diagnosis of ALK+ LBCL is challenging due to the rare incidence, morphology resembling DLBCL, plasma cell neoplasms, myeloid sarcomas, and epithelial neoplasms, and ALK immunostain is not routinely performed in practice. Key differential diagnoses to be considered include ALK-positive anaplastic large cell lymphoma (ALK+ ALCL), plasmacytoma, immunoblastic/plasmablastic variants of DLBCL, plasmablastic lymphoma (PBL), and primary effusion lymphoma (PEL). ALK+ LBCL and ALK+ ALCL can show significant overlap in terms of clinical presentation, sinusoidal pattern of involvement, and markers such as CD45, EMA, and CD4. One important distinguishing feature is cytology where ALK+ LBCL shows plasmablastic morphology and ALK+ ALCL commonly shows anaplastic morphology with “hallmark cells.” Additionally, CD30 is usually positive in ALK+ ALCL and negative in ALK+ LBCL. ALK+ LBCL can also share many similarities with other B-cell lymphomas such as PBL and PEL in terms of cytology and immunophenotypic features including expression of plasma cell markers and lack of conventional B-cell markers. PBL and PEL commonly occur in HIV-positive patients and are associated with EBV and HHV8, respectively. Other variants of DLBCL are less difficult to distinguish from ALK+ LBCL due to the presence of common B-cell markers such as CD20 and PAX5 and the lack of ALK expression. Plasmacytoma (primary bone and extraosseous forms), including morphologic variants such as anaplastic plasmacytoma, can often resemble ALK+ LBCL. There is significant immunophenotypic overlap including' expressions of plasmacytic differentiation. However, features including the presence of bone involvement, myeloma component, and ALK immunohistochemistry can help distinguish plasmacytoma from ALK+ LBCL. In the workup of a suspected case of ALK+ LBCL, the plasmblastic morphology/immunophenotype (CD38 and CD138), lack of CD30, expression of ALK, and the lack of confirmed viral associations (EBV, HHV8, and HIV) would aid the diagnosis of ALK+ LBCL.

Our case demonstrates the classic morphology and immunophenotypic profile of ALK+ LBCL but with aberrant CD33 expression. Since CD33 is not a commonly used marker in lymphoma workup, the rationale for its use in this case was based on the broad initial differential diagnosis, which included myeloid sarcoma and ALK+ ALCL. To the best of our knowledge, myeloid lineage expression has not been ever reported in the literature. In a fairly recent, large series study of ALK+ LBCL with literature review by Pan et al., myeloid lineage-associated markers were not reported [2]. Given the unusual CD33 expression, we performed NGS panel looking for common myeloid malignancy-associated mutations; however, the results were negative, and the significance of myeloid expression in our case is uncertain. MPO was also performed on the case and was negative. Although CD33 expression was an incidental finding in our case, it illustrates the fact that ALK+ LBCL is a rare entity, and when initial immunohistochemistry panels for T and B cells proved to be inconclusive, the differential diagnosis can expand to hematologic neoplasm of other lineages. Finally, the clinical significance of this finding is unknown.

In conclusion, we present a rare case of ALK+ LBCL with unusual CD33 expression that has not been reported in the literature. The pathologic significance of CD33 expression is unclear based on the lack of myeloid-associated mutations by molecular studies and whether there is a clinical significance is also uncertain. To the best of our knowledge, the patient is currently alive and undergoing therapy.
